# Tyrosinase Inhibitors from the Aerial Parts of *Wulfenia carinthiaca* jacq.

**DOI:** 10.1002/cbdv.201800014

**Published:** 2018-04-17

**Authors:** Benjamin Mutschlechner, Bettina Rainer, Stefan Schwaiger, Hermann Stuppner

**Affiliations:** ^1^ Institute of Pharmacy/Pharmacognosy Center for Molecular Biosciences Innsbruck (CMBI) Center for Chemistry and Biomedicine University of Innsbruck Innrain 80‐82 Innsbruck 6020 Austria; ^2^ MCI Management Center Innsbruck Maximilianstraße 2 Innsbruck 6020 Austria

**Keywords:** *Wulfenia carinthiaca*, Plantaginaceae, mushroom tyrosinase inhibition (oxidoreductase), iridoid glycosides, phenylethanoid glucosides

## Abstract

Activity guided isolation of a MeOH extract of the aerial plant parts of *Wulfenia carinthiaca* jacq. (Plantaginaceae), using a mushroom tyrosinase assay, resulted in the isolation of five phenylethanoid glucosides and four iridoid glycosides. Two of them, 2′‐*O*‐acetylisoplantamajoside and 2′,6″‐*O*‐diacetylisoplantamajoside, represent new natural products. Evaluation of the inhibitory activity of all isolated compounds revealed that the observed activity is not related to the isolated phenylethanoid glycosides but mainly due to the presence of the iridoid glycoside globularin (*IC*_50_ 41.94 μm;*CI*_95%_ ± 16.61/11.89 μm). Interestingly, structurally close related compounds (globularicisin, baldaccioside, and isoscrophularioside) showed no or only a weak tyrosinase inhibitory activity.

## Introduction

The genus *Wulfenia* (Plantaginaceae) belongs to a group of genera with a disjunct distribution range and is currently classified into four species s.str.: *Wulfenia carinthiaca* s.l. (Carnic and the Dinaric Alps populations), *W*. *baldacii* (restricted to the Dinaric alps), *W*. *orientalis* (restricted to the southernmost Amanos Mts., Turkey), and *W*. *glanduligera* (central and northern Amanos Mts., Turkey).^1^ Due to the limited local distribution and the attractive intensive blue color of the flowers, especially of *Wulfenia carinthiaca*, this species was selected as the national flower of the federal state of Carinthia/Austria.^2^

Nowadays, also a white flowering cultivar, *W*. *carinthiaca* cv. ‘Alba’ is commercially available from different providers. Despite its popularity as ornamental plant, the phytochemical knowledge about the genus is limited. An investigation of the root material of *W*. *carinthiaca* resulted in the identification of the phenylethanoids plantamajoside, 2′‐*O*‐acetylplantamajoside, and 2′,6″‐*O*‐diacetylplantamajoside, as well as the irdoides globularin, isoscrophularoside, and the dimeric iridoid wulfenoside.^3^ In a comparative chemotaxonomic study of tribe Veroniceae (Plantaginaceae)^4^ also other representatives (whole plant samples) were investigated: *W*. *baldaccii* afforded mannitol, plantamajoside, 2′,6″‐*O*‐diacetylplantamajoside, catalpol and asystasioside E, epiloganic acid, aucubin, globularin, isoscrophularioside, picroside I, wulfenoside, and the new chlorine containing iridoid baldaccioside; *W*. *orientalis* contained mannitol and shikimic acid, catalpol, gardoside, aucubin, mussaenosidic acid, arborescosidic acid, globularin, isoscrophularioside, as well as the phenylethanoide 2′,6″‐*O*‐diacetylplantamajoside; *Wulfenia blechicii* subsp. *rohleanae*, a ‘species’ which is distributed only in the Dinaric Alps and should be nowadays handled as *W*. *carinthiaca*, since the species status is neither supported by genetic nor by morphologic data.^1^ Nevertheless, the investigation of the plant material afforded shikimic acid, catalpol, 6,7‐dihydromonotropein, aucubin, gardoside, epiloganic acid and arborescosidic acid, *cis*‐globularin (= globularicisin), globularin, picroside, and isoscrophularioside. The MeOH extract of the aerial parts of *W*. *carinthiaca* (Carnic Alps population, cultivated material) showed in an HPTLC‐based mushroom tyrosinase inhibition assay^5^ a promising inhibitory effect, which was also evaluated in a 96 well based assay. In this assay, the MeOH extract showed an inhibitory effect of *ca*. 40% at a concentration of 500 μg/mL (highest soluble concentration). Tyrosinase, a copper‐containing mono‐oxygenase enzyme, catalyzes the first two steps of the melanogenesis, converting firstly l‐tyrosine by hydroxylation to 3,4‐dihydroxyphenylalanine and subsequently by oxidation to dopaquinone.^6^ Inhibitors of this enzyme, especially those without side effects, are of interest in cosmetic industry to reduce undesired pigmentation like freckles and age spots.

In this connection, two questions arise: Is a differentiation of the two populations of *W*. *carinthiaca* (Carnic and Dinaric Alps) based on the secondary metabolite pattern possible and what compound(s) is/are responsible for the observed tyrosinase inhibition?

## Results and Discussion

**Figure 1:**
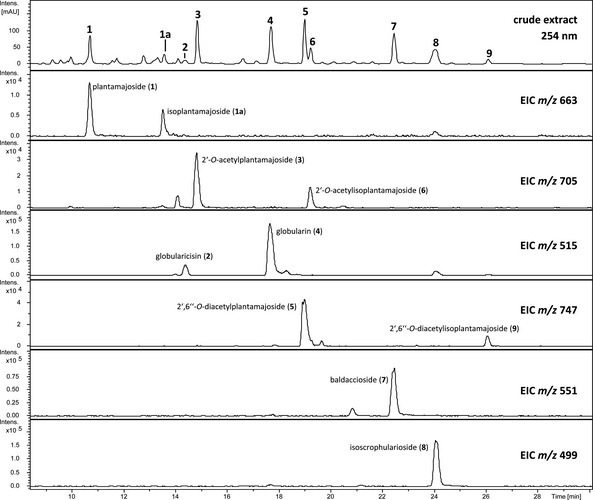
Results of the LC/MS analysis of the MeOH extract of *W*. *carinthiaca* [5 mg/mL] at 254 nm as well as individual extracted ion chromatograms (ESI, positive‐ion mode) representing the sodium adduct ions [*M* + Na]^+^ of compound **1** – **9**.

**Figure 2:**
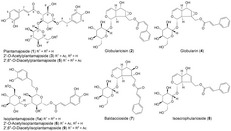
Structures of isolated or tentatively identified (compound **1a**) phenylethanoide and iridoid glucosides of the aerial parts of *W*. *carinthiaca*.

In a first step, the obtained MeOH extract of the aerial plant parts of *W*. *carinthiaca* was investigated by LC/MS (see *Figure* 1 and *Table* 1). Taking into consideration the constituents already described for the roots of *W*. *carinthiaca* and other species of this genus and analyzing the extracted ion chromatograms (ESI, positive‐ion mode) of the corresponding sodium adduct ions ([*M* + Na]^+^) the presence of the iridoid glucosides globularicisin (**2**), globularin (**4**), baldaccioside (**7**), and isoscrophularoside (**8**) (see *Figure* 2) could be suggested.^3^, ^4^

**Table 1:** LC/MS data (LC/ESI‐MS) of identified and tentatively assigned (**1a**) compounds of the MeOH extract of the aerial parts of *W*. *carinthiaca* with proposed fragments

Compound	*t*_R_ [min]	*m/z* Positive‐ion mode [rel. int. %]	*m/z* Negative‐ion mode [rel. int. %]
Plantamajoside (**1**)	10.7	663.0 (3.1, [*M* + Na]^+^), 658.0 (1.8, [*M* + NH_4_]^+^), 478.8 (9.7, [*M* – caffeoyl]^+^), 324.9 (100, [*M* – caffeoyl – 3‐OH‐tyrosol]^+^), 163.1 (75.3, [caffeoyl]^+^)	638.9 (100, [*M* – H]^−^)
Isoplantamajoside (**1a**)	13.5	663.0 (6.4, [*M* + Na]^+^), 657.9 (1.8, [*M* + NH_4_]^+^), 478.8 (43.8, [*M* – caffeoyl]^+^), 324.9 (100, [*M* – caffeoyl – 3‐OH‐tyrosol]^+^), 163.1 (90.3, [caffeoyl]^+^)	638.9 (100, [*M* – H]^−^)
Globularicisin (= *cis*‐globularin, **2**)	14.4	514.9 (86.9, [*M* + Na]^+^), 492.8 (27.2, [*M* + H]^+^), 478.8 (71.5), 324.9 (100), 330.9 (100, [*M* – glucose]^+^)	n.d.
2′‐*O*‐Acetylplantamajoside (**3**)	14.8	705.0 (4.5, [*M* + Na]^+^), 700.0 (2.9, [*M* + NH_4_]^+^), 683.1 (0.4, [*M* + H]^+^), 366.8 (100, [*M* – caffeoyl – 3‐OH‐tyrosol]^+^), 163.1 (60.6, [caffeoyl]^+^)	681.1 (100, [*M* – H]^−^)
Globularin (**4**)	17.6	514.9 (54.8, [*M* + Na]^+^), 493.0 (97.2, [*M* + H]^+^), 331.0 (100, [*M* – glucose]^+^)	n.d.
2′,6″‐*O*‐Diacetylplantamajoside (**5**)	19.0	747.1 (5.8, [*M* + Na]^+^), 742.0 (3.7, [*M* + NH_4_]^+^), 570.8 (3.5, [*M* – 3‐OH‐tyrosol]^+^), 381.9 (8.24), 366.9 (100, [*M* – caffeoyl – 3‐OH‐tyrosol – acetate]^+^), 163.1 (60.6, [caffeoyl]^+^)	723.0 (100, [*M* – H]^−^)
2′‐*O*‐Acetylisoplantamajoside (**6**)	19.2	705.0 (6.0, [*M* + Na]^+^), 528.9 (11.5, [*M* – 3‐OH‐tyrosol]^+^), 366.9 (100, [*M* – caffeoyl – 3‐OH‐tyrosol]^+^), 163.1 (99.5, [caffeoyl]^+^)	680.9 (100, [*M* – H]^−^)
Baldaccioside (**7**)	22.4	551.0 (100, [*M* + Na]^+^), 292.9 (54.5), 131.2 (91.5)	572.8 (48.1, [*M* + formate]^−^), 562.8 (22.5, [*M* + chloride]^−^), 527.0 (100, [*M* – H]^−^)
Isoscrophularoside (**8**)	24.0	499.0 (100, [*M* + Na]^+^), 458.9 (18.2, [*M* – H_2_O + H]^+^), 296.9 (26.2), 279.0 (63.7), 131.2 (34.3)	520.7 (100, [*M* + formate]^−^), 474.7 (18.3, [*M* – H]^−^)
2′,6″‐*O*‐Diacetylisoplantamajoside (**9**)	26.1	747.0 (6.7, [*M* + Na]^+^), 570.9 (18.8, [*M* – 3‐OH‐tyrosol]^+^), 381.9 (29.0), 366.9 (100, [*M* – caffeoyl – 3‐OH‐tyrosol – acetate]^+^), 163.1 (57.6, [caffeoyl]^+^)	722.9 (100, [*M* – H]^−^)

This was confirmed by the isolation of the corresponding compounds and analysis of the recorded 1D‐ and 2D‐NMR spectra. The LC/MS analysis revealed also the presence of several phenylethanoid glycosides in the extract of the aerial plant parts (see *Figure* 2) corresponding to plantamajoside and its derivatives (**1**,** 3**, and **5**) known from the roots of *W*. *carinthiaca*.^3^ Interestingly, all three compounds showed in the EIC an additional peak with an identical LC‐online UV spectrum and identical *m/z* values corresponding to the sodium adduct ion in the positive‐ion mode ESI‐MS and the deprotonated molecule ion ([*M* − H]^−^) in the negative‐ion mode ESI‐MS. Differences were only observed in the intensity of some of the detected fragments in the positive‐ion mode ESI‐MS (see *Table* 1). The MS spectra of compound **1** and **1a** showed *e.g*. a significant difference in the relative intensity (9.7% rel. intensity (**1**) and 43.8% rel. intensity (**1a**)) of a fragment with an *m/z* of 478.8, suggesting a very similar, but not identical structure of both compounds. The compound pairs **3** and **6**, as well as **5** and **9** showed an analogous behavior. In order to elucidate the chemical nature of the additional phenylethanoid glycosides, **6** and **9** were isolated together with **1**,** 3**, and **5**. All compounds could be obtained in a satisfying purity and quantity for NMR structure elucidation except compound **1a**.

Comparison of the NMR spectra of compounds **3** and **6** with literature values enabled the identification of compound **3** as 2′‐*O*‐acetylplantamajoside.^3^ Careful analysis of the HMBC contacts of the protons attached to the carbons 4′ and 6′ of the inner glucose moiety of compound **6** revealed a different connectivity of the caffeoyl moiety, which was found to be shifted from position 4′ in compound **3** to position 6′ of the inner glucose subunit in compound **6**. The change of the ester position is therefore identical to the well‐known pair acteoside/isoacteoside and was already described for plantamajoside and the related compound isoplantamajoside,^7^ also known as plantainoside D.^8^ Therefore, compound **6** was identified as 2′‐*O*‐acetylisoplantamajoside. Comparison of the NMR spectra of compounds **5** and **9** resulted in an analogous result. Compound **5** could be identified as 2′,6″‐*O*‐diacetylplantamajoside, while compound **9** was identified as 2′,6″‐*O*‐diacetylisoplantamajoside. Both isolated acetylated isoplantmajoside derivatives are described here for the first time.

Due to the pairwise occurrence of plantamajoside and isoplantamajoside derivatives we tentative identified compound **1a** as isoplantamajoside, which is supported by the observed LC/ESI‐MS fragments showing slightly elevated levels of liberated caffeoyl units as well as the remaining molecule parts similar to the NMR‐verified pairs **3** and **6** or **5** and **9**. From a chemotaxonomic point of view it is interesting that a differentiation of the two populations of *W*. *carinthiaca* (Carnic and Dinaric Alps) seems to be possible, since *W*. *carinthiaca* from the Dinaric Alps (previous described as *Wulfenia blechicii* subsp. *rohleanae*) seems to contain no phenylethanoids at all.^4^ For final confirmation of the chemotaxonomic relevance of these findings, results have to be verified with a larger number of samples which should also include wild type specimens.

**Figure 3:**
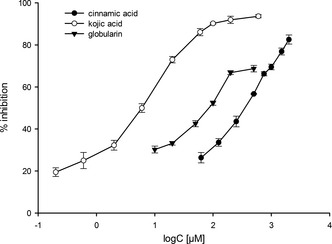
Concentration‐response curves of cinnamic acid, kojic acid, and globularin in the tyrosinase inhibition assay. Results are shown as mean of *n* = 3 ± SD.

In order to assess which compound(s) of *W*. *carinthiaca* is/are responsible for the observed inhibitory effect on mushroom tyrosinase, compounds **1** – **9** were evaluated at a concentration of 500 μm in the 96‐well plate assay. The results are summarized in *Table* 2. Since only the iridoid globularin (**4**, see *Figure* 2) showed a promising effect at this rather high test concentration, *IC*_50_ value determination was limited to this compound together with free cinnamic acid, a known tyrosinase inhibitor,^9^ and kojic acid as positive control (see *Figure* 3).

**Table 2:** Evaluation of the tyrosinase inhibition of the main constituents of *W*. *carinthiaca* at a concentration of 500 μm (*n* = 3). Positive control: kojic acid (*IC*_50_ = 5.40 μm;* CI*_95%_ ± 0.47/0.43 μm)

Compound	Inhibitory activity (given in % inhibition ± SD)
Plantamajoside (**1**)	0.11 ± 3.61
Globularicisin (= *cis*‐globularin; **2**)	4.20 ± 6.06
2′‐*O*‐Acetylplantamajoside (**3**)	33.07 ± 1.00
Globularin (**4**)	79.59 ± 1.62
2′,6″‐*O*‐Diacetylplantamajoside (**5**)	29.76 ± 4.24
2′‐*O*‐Acetylisoplantamajoside (**6**)	13.50 ± 3.10
Baldaccioside (**7**)	23.01 ± 3.16
Isoscrophularoside (**8**)	48.49 ± 2.08
2′,6″‐*O*‐Diacetylisoplantamajoside (**9**)	26.14 ± 3.18

Globularin showed in the tyrosinase inhibition assay an *IC*_50_ value of 41.94 μm (*CI*_95%_ ± 16.61/11.89 μm) and is therefore not as active as the positive control kojic acid (*IC*_50_ of 5.40 μm (*CI*_95%_ ± 0.47/0.43 μm). Interestingly, free cinnamic acid shows significant higher *IC*_50_ value of 353.99 μm (*CI*_95%_ ± 27.24/25.29 μm). Accordingly, it can be assumed that the iridoid part of globularin improves the interaction with the enzyme. Since the *cis*‐isomer of globularin, globularicisin (**2**), showed almost no activity at 500 μm, the *trans*‐configuration of the cinnamic acid unit appears to be essential for the inhibitory activity, but also the shape and substitution pattern of the iridoid part affects the activity. Replacement of the epoxide ring of **4** by a double bond (isoscrophularioside, **8**) reduces the inhibitory effect from 79.59 ± 1.62% to 48.49 ± 2.08% at 500 μm. The activity reduction is even more evident when the epoxide ring is opened by the addition of hydrogen chloride to the molecule, which led to a drop of activity from 79.59 ± 1.62% (**4**) to 23.01 ± 3.16% (**7**) at 500 μm.

## Conclusions

In summary, phytochemical investigation of the aerial parts of *Wulfenia carinthiaca* resulted in the isolation and identification of four iridoid glycosides and five phenylethanoid glycosides, of which two, 2′‐*O*‐acetylisoplantamajoside (**6**) and 2′,6″‐*O*‐diacetylisoplantamajoside (**9**), are described for the first time. Detection of phenylethanoid glycosides in the investigated plant parts might be useful for the chemical differentiation of the two populations of *W*. *carinthiaca* (Carnic and Dinaric Alps). Furthermore, the pharmacological evaluation of isolated compounds led to the identification of the novel tyrosinase inhibitor globularin.

## Experimental Section

### General

Solvents and Reagents: all used solvents were provided by *VWR International* (Darmstadt, Germany). Solvents used for HPLC analysis were obtained from *Merck* (Darmstadt, Germany). Ultrapure water, for the HPLC analysis, was produced by a *Sartorius Arium 611 UV* water purification system (*Sartorius AG*, Göttingen, Germany). Solvents used for NMR spectroscopy were purchased from *Euriso‐top SAS* (Saint‐Aubin Cedex, France). Mushroom tyrosinase, l‐DOPA and kojic acid used for the tyrosinase inhibition assay were purchased from *Sigma–Aldrich* (St. Louis, Missouri, USA). LC Method: for the analysis of the extract, its fractions and single compounds an *Agilent 1100* series HPLC system (*Agilent*, Waldbronn, Germany) equipped with autosampler, column thermostat, on‐line degasser, quaternary pump and DAD was used. Stationary phase: *YMC* (Kyoto, Japan) *Pack Pro C*_*18*_ column (150 × 4.6 mm i.d., 3.0 μm particle size). Solvent *A* consisted of ultrapure water containing 0.9% formic acid and 0.1% acetic acid, and solvent *B* consisted of acetonitrile. The flow rate was set to 1.0 mL/min. Solvent gradient: 0 min 88% *A*; 30 min to 70% *A*; 33 min 2% *A*; 45 min stop; post time: 10 min; column oven temperature: 45 °C, injection volume: 5 μL. The detection wavelength was set to 254 nm. LC/MS and HR‐MS methods: LC/MS and tandem mass spectrometry was performed on an *Agilent 1100* series system (*Agilent*, Waldbronn, Germany) equipped with autosampler, column thermostat, on‐line degasser, quaternary pump and DAD, coupled with *Esquire 3000Plus* (*Bruker Daltonics*, Bremen, Germany) and on an *Agilent 1200* series HPLC system, equipped with DAD, autosampler, online degasser, column thermostat and quaternary pump, coupled with *Bruker mikrOTOF‐QII*. HPLC settings and gradient elution were selected as mentioned before. The MS parameters for the *Esquire 3000Plus* (*Bruker Daltonics*, Bremen, Germany) were: split, 1:5; ESI, negative/positive‐ion mode; spray voltage, 4.5 kV; dry temperature, 300 °C; drying gas flow rate, 10.00 L/min; nebulizer gas, 30 psi; mode, scan range: *m/z* 100 – 1200. The MS parameters for the *Bruker mikrOTOF‐QII* were the following: split 1:0.4; negative/positive‐ion mode ESI; scan range, *m/z* 50 – 1500 with a scan rate of 2 Hz; nebulizer gas 23.2 psi; dry gas 8.0 L/min at a temperature of 220 °C; capillary energy 3000 V; funnel 1 RF 200.0 Vpp; funnel 2 RF 200 Vpp; hexapole RF 200.0 Vpp; quadrupole ion energy 5 eV; collision energy 15 eV; transfer time 90 μs; collision RF 165 Vpp; prepulse storage 8 μs. High‐resolution mass calibration was facilitated by injecting 10 mm NaOH in isopropanol/water (1:1, v/v) fortified with 0.1% formic acid at the beginning of each analysis. The exact mass of the sodium formate clusters were used for internal calibration. Polarimeter: for the determination of the optical rotation a *PerkinElmer Polarimeter 341* was used. Melting point: the melting point was determined at a *Reichert* heating microscope. NMR: 1D‐ and 2D‐NMR experiments were recorded on a *Bruker Advance II* (*Bruker Biospin Rheinstetten*, Germany) 600 NMR spectrometer. Semi‐preparative HPLC: Semi‐preparative HPLC was carried out on a *Dionex* preparative HPLC system (*P580* pump, *ASI 100* automated sampler, *Ultimate 3000* column department, *UVD 170 U* detector; Dionex Softron, Germerling, Germany) equipped with a *Gillson 206* fraction collector.

### Plant Material

Plant material of *W*. *carinthiaca* used for the preparation of the MeOH extract and isolation was cultivated by the Botanical Garden in Innsbruck and collected in September 2001 (voucher specimen: MD01‐2319),^3^ and in July 2014 (voucher specimen: BW 20140717‐30).

### Extraction and Isolation

150.7 g of the air dried plant material (aerial plant parts; batch MD01‐2319) were milled and exhaustively extracted by maceration at room temperature with methanol. After solvent evaporation (35 °C/rotary evaporator), 60.0 g of a dark green extract were obtained. The extract was stored at −20 °C until further processing. Due to the long time period between extract preparation and processing, a fresh extract (batch BW 20140717‐30) was prepared. Both extracts were compared by means of HPLC, resulting in the detection of quantitative, but not qualitative differences of both extracts (see *Supporting Information*, Figures S1andS2). For further isolation 16.01 g of the MeOH extract (batch MD01‐2319) were fractionated by means of liquid‐liquid separation. Therefore, the extract was suspended in 500 mL HPLC water and exhaustively (until colorlessness of the organic layer) extracted with 500 mL portions of petroleum ether (yield: 0.22 g), diethyl ether (yield: 1.15 g), ethyl acetate (yield: 5.26 g). A part of the obtained ethyl acetate fraction (550.0 mg) was dissolved in methanol and separated by *Sephadex LH20* column chromatography (gel bed dimension: 880 × 35 mm, mobile phase methanol). The eluate was collected in portions of 2 mL and analyzed with TLC: ethyl acetate/methanol/water/formic acid (77:15:8:1; all v/v); derivatization: anisaldehyde/H_2_SO_4_ reagent, 120 °C. Fractions with similar compositions were combined to a total of 14 fractions, *Frs. WE‐S1* – *WE‐S14*. *Fr. WE‐S12* (74.3 mg) consisted of the main constituent of the extract, which was identified by means of 1‐ and 2D‐NMR as 2′,6″‐*O*‐diacetylplantamajoside (**5**).^3^ *Fr. WE‐S8* (88.7 mg) was separated with semi‐preparative HPLC: stationary phase *Phenomenex Aqua C*_*18*_ column; 5 μm, 125 Å, 250 × 10.0 mm with corresponding guard column; mobile phases: water (*A*) and acetonitrile (*B*); flow rate: 2.0 mL/min; solvent gradient: 0 min 70% *A*; 25 min 2% *A*; post time: 10.0 min; column oven temperature: 40 °C; injection volume: 10 μL of a solution of 29.6 mg/mL per run; detection wavelength: 254 nm. Fractionation time frame: 12.00 – 12.70 min *cis*‐globularin (**2**),^10^ 14.30 – 15.00 min globularin (**4**),^10^ 17.40 – 18.00 min baldaccioside (**7**),^4^ and 19.70 – 21.50 min isoscrophularioside (**8**).^11^ *Fr. WE‐S14* (120.5 mg) was also separated with semi‐preparative HPLC: stationary phase *Phenomenex Aqua C*_*18*_ column; 5 μm, 125 Å, 250 × 10.0 mm with corresponding guard column; mobile phases: water (*A*) and acetonitrile (*B*); flow rate: 2.0 mL/min; solvent gradient: start 90% *A*; 24.0 min 55.4% *A*; 25.0 min 2% *A*; 30.0 min 2% *A*; post time: 10.0 min; column oven temperature: 35 °C; injection volume: 50 μL of a solution of 30 mg/mL per run; detection wavelength: 254 nm. Fractionation time frame: 16.18 – 17.36 min plantamajoside (**1**),^7^ 18.00 – 18.30 min, 2′‐*O*‐acetylplantamajoside (**3**),^3^ 18.50 – 19.45 min 2′‐*O*‐acetylisoplantamajoside (**6**) with traces of (**5**) and 22.36 – 23.42 min 2′,6″‐*O*‐diacetylisoplantamajoside (**9**).

**2**′**‐** ***O*** **‐Acetylisoplantamajoside** (= **2‐(3,4‐Dihydroxyphenyl)ethyl 4‐** ***O*** **‐Acetyl‐6‐** ***O*** **‐[(2** ***E*** **)‐3‐(3,4‐dihydroxyphenyl)prop‐2‐enoyl]‐3‐** ***O*** **‐** ***β*** **‐** **d** **‐glucopyranosyl‐** ***β*** **‐** **d** **‐glucopyranoside**;** 6**). M.p. 205 °C. ${\left[\alpha \right]}_{D}^{20}$ = −32.27 (*c* = 0.11, MeOH). ^1^H‐NMR (600.2 MHz, CD_3_OD): 3‐hydroxytyrosol moiety: 6.63 (*d*,* J* = 2.0, H–C(2)); 6.64 (*d*,* J* = 8.1, H–C(5)); 6.50 (*dd*,* J* = 8.0, 2.0, H–C(6)); 2.66 – 2.71 (*m*, CH_2_(7)); 4.00 (*ddd*,* J* = 9.8, 6.7, 5.9, H_a_–C(8)); 3.61 – 3.64 (*m*, H_b_–C(8)); caffeoyl moiety: 7.08 (*d*,* J* = 2.0, H–C(2)); 6.77 (*d*,* J* = 8.2, H–C(5)); 6.92 (*dd*,* J* = 8.2, 2.0, H–C(6)); 7.56 (*d*,* J* = 15.9, H–C(7)); 6.27 (*d*,* J* = 15.9, H–C(8)); internal glucose moiety: 4.49 (*d*,* J* = 8.1, H–C(1′)); 4.85 – 4.89 (*m*, H–C(2′)); 3.62 – 3.67 (*m*, H–C(3′)); 3.70 – 3.73 (*m*, H–C(4′)); 3.50 – 3.53 (*m*, H–C(5′)); 4.54 (*dd*,* J* = 11.8, 2.0, H_a_–C(6′)); 4.32 – 4.37 (*m*, H_b_–C(6′)); terminal glucose moiety: 4.31 – 4.36 (*m*, H–C(1″)); 3.17 (*dd*,* J* = 8.9, 8.0, H–C(2″)); 3.29 – 3.35 (*m*, H–C(3″)); 3.24 – 3.29 (*m*, H–C(4″)); 3.15 – 3.21 (*m*, H–C(5″)); 3.86 (*dd*,* J* = 11.8, 2.0, H_a_–C(6″)); 3.67 – 3.69 (*m*, H_b_–C(6″)); 2′*‐O‐*acetyl moiety: 1.97 (*s*, Me). ^13^C‐NMR (150.9 MHz, CD_3_OD): 3‐hydroxytyrosol moiety: 131.68 (C); 117.15 (CH); 146.80 (C); 144.58 (C); 116.55 (CH); 121.29 (CH); 36.41 (CH_2_); 71.92 (CH_2_); caffeoyl moiety: 127.68 (C); 115.12 (CH); 146.02 (C); 149.66 (C); 116.55 (CH); 123.13 (CH); 147.27 (CH); 114.80 (CH); 169.02 (C); internal glucose moiety: 102.11 (CH); 73.70 (CH); 85.15 (CH); 70.41 (CH); 75.15 (CH); 64.41 (CH_2_); terminal glucose moiety: 105.33 (CH); 74.68 (CH); 78.07 (CH); 71.39 (CH); 77.94 (CH); 62.51 (CH_2_); 2′*‐O‐*acetyl moiety: 172.07 (C); 21.10 (Me). LC/HR‐MS: 681.2142 ([*M* – H]^−^, $C_{31}H_{37}O_{17}^{-}$; calc. 681.2036).

**2**′**,6**″**‐** ***O*** **‐Diacetylisoplantamajoside** (= **2‐(3,4‐Dihydroxyphenyl)ethyl 4‐** ***O*** **‐Acetyl‐3‐** ***O*** **‐(6‐** ***O*** **‐acetyl‐** ***β*** **‐** **d** **‐glucopyranosyl)‐6‐** ***O*** **‐[(2** ***E*** **)‐3‐(3,4‐dihydroxyphenyl)‐prop‐2‐enoyl]‐** ***β*** **‐** **d** **‐glucopyranoside**;** 9**). M.p. 240 °C. ${\left[\alpha \right]}_{D}^{20}$ = −8.89 (*c* = 0. 19, MeOH). ^1^H‐NMR (600.2 MHz, CD_3_OD): 3‐hydroxytyrosol moiety: 6.64 (*d*,* J* = 2.0, H–C(2)); 6.62 (*d*,* J* = 8.0, H–C(5)); 6.50 (*dd*,* J* = 8.0, 2.0, H–C(6)); 2.65 – 2.71 (*m*, H_a_–C(7)); 2.66 – 2.71 (*m*, H_b_–C(7)); 4.00 (*dt*,* J* = 9.6, 6.3, 6.3, H_a_–C(8)); 3.61 – 3.65 (*m*, H_b_–C(8)); caffeoyl moiety: 7.04 (*d*,* J* = 2.0, H–C(2)); 6.78 (*d*,* J* = 8.2, H–C(5)); 6.93 (*dd*,* J* = 8.2, 2.0, H–C(6)); 7.56 (*d*,* J* = 15.8, H–C(7)); 6.29 (*d*,* J* = 15.8, H–C(8)); internal glucose moiety: 4.51 (*d*,* J* = 8.1, H–C(1′)); 4.86 (*dd*,* J* = 8.1, 9.6, H–C(2′)); 3.63 – 3.69 (*m*, H–C(3′)); 3.48 (*dd*,* J* = 8.9, 9.5, H–C(4′)); 3.59 – 3.63 (*m*, H–C(5′)); 4.53 (*dd*,* J* = 11.9, 2.1, H_a_–C(6′)); 4.32 – 4.36 (*m*, H_b_–C(6′)); terminal glucose moiety: 4.33 (*d*,* J* = 11.9, H–C(1″)); 3.57 (*ddd*,* J* = 9.5, 7.1, 2.3, H–C(2″)); 3.28 (*d*,* J* = 9.5, H–C(3″)); 3.34 (*d*,* J* = 9.1, H–C(4″)); 3.16 – 3.21 (*m*, H–C(5″)); 4.14 (*dd*,* J* = 11.9, 7.1, H_a_–C(6″)); 4.44 (*dd*,* J* = 11.9, 2.8, H_b_–C(6″)); 2′*‐O‐*acetyl moiety: 1.97 (*s*, Me); 6″*‐O‐*acetyl moiety: 2.05 (*s*, Me). ^13^C‐NMR (150.9 MHz, CD_3_OD): 3‐hydroxytyrosol moiety: 131.69 (C); 117.16 (CH); 146.82 (C); 144.61 (C); 116.27 (CH); 121.29 (CH); 36.43 (CH_2_); 71.94 (CH_2_); caffeoyl moiety: 127.71 (C); 115.12 (CH); 146.05 (C); 149.66 (C); 116.54 (CH); 123.13 (CH); 147.27 (CH); 114.82 (CH); 169.00 (C); internal glucose moiety: 102.15 (CH); 73.27 (CH); 86.76 (CH); 70.48 (CH); 75.11 (CH); 64.80 (CH_2_); terminal glucose moiety: 105.61 (CH); 75.29 (CH); 71.69 (CH); 77.77 (CH); 74.53 (CH); 64.37 (CH_2_); 2′*‐O‐*acetyl moiety: 172.74 (C); 21.04 (Me); 6″*‐O‐*acetyl moiety: 172.06 (C); 20.65 (Me). LC/HR‐MS: 723.2136 ([*M* − H]^−^, $C_{33}H_{39}O_{18}^{-}$; calc. 723.2142).

### Tyrosinase Inhibition Assay

Tyrosinase inhibitory activity of the extract or pure compounds was evaluated by a dopachrome method using l‐DOPA as a substrate. The 96‐well microplate method was used with slight modifications.^12^ Each concentration of the sample solution were denoted as *A* (three wells), as *B* (one well), as *C* (three wells), and as *D* (one well), which contained following mixture (200 μL): *A*: 80 μL of a 1/15 m phosphate buffer (pH 6.8), 40 μL 1/15 m phosphate buffer (pH 6.8) containing 5% DMSO (solvent control) and 40 μL of mushroom tyrosinase (100 units/mL) dissolved in 1/15 m phosphate buffer (pH 6.8). *B* contained 120 μL 1/15 m phosphate buffer (pH 6.8) and 40 μL solvent control, *C* 80 μL 1/15 m phosphate buffer (pH 6.8), 40 μL of mushroom tyrosinase (100 units/mL) in the same buffer and 40 μL of the sample‐buffer solution containing 5% DMSO to dissolve the sample. *D* consisted of 120 μL phosphate buffer and 40 μL of sample solution containing 5% DMSO. Kojic acid (*IC*_50_ 5.4 μm) was used as positive control. Each well was mixed and incubated at 23 °C for 10 min. Then 40 μL of 0.3 mm l‐DOPA in the same buffer was added. After 5 min of incubation at 23 °C, the absorbance at 475 nm in each well was measured using a *TECAN Spark 10M* microplate photometer (*Tecan Group AG*, Männerdorf, Switzerland). The percentage inhibition of tyrosinase activity was calculated by equation: {[(*A* – *B*) – (*C* – *D*)]/(*A* – *B*)} × 100. *IC*_50_ values were determined using GraphPad Prismn 5.

## Author Contribution Statement

*H. S*. and *S. S*. conceived and designed the experiments; *B. M*. and *B. R*. performed the experiments; *B. M*., *B. R*., and *S. S*. analyzed the data; *B. M*., *S. S*., and *H. S*. wrote the article.

## Supporting information

Supporting information for this article is available on the WWW under https://doi.org/10.1002/cbdv.201800014.
