# Supplementary material for: Tyrosinase Inhibitors from the Aerial Parts of Wulfenia carinthiaca jacq
Source: Chem Biodivers. 2018 Apr 17;15(4):e1800014. doi: 10.1002/cbdv.201800014 (PMC6468267; doi:10.1002/cbdv.201800014)
Supplement: Supplementary file 1 [file CBDV-15-na-s001.docx]

Tyrosinase Inhibitors from the Aerial Parts of *Wulfenia carinthiaca* Jacq.

Benjamin Mutschlechner,^a^ Bettina Rainer,^a,b^ Stefan Schwaiger,^a,^* and Hermann Stuppner^a^

^a^ Institute of Pharmacy/Pharmacognosy, University of Innsbruck and Center for Molecular Biosciences Innsbruck (CMBI), Center for Chemistry and Biomedicine, Innrain 80-82, 6020 Innsbruck, Austria, e-mail stefan.schwaiger@uibk.ac.at

^b^ MCI Management Center Innsbruck, Maximilianstraße 2, 6020 Innsbruck, Austria


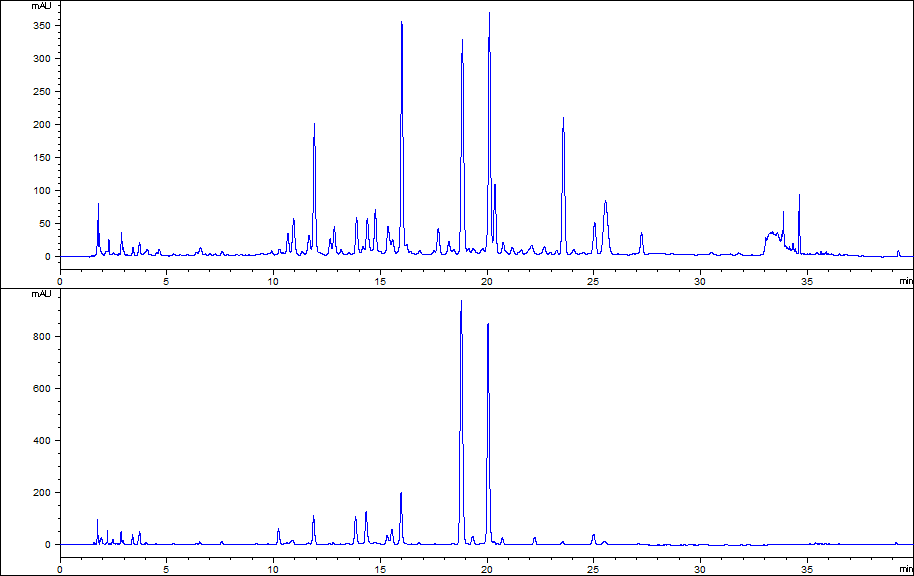


**Figure S1.** HPLC-comparison of methanolic extracts of the aerial plant parts of *W. carinthiaca* voucher specimen MD01-2319 (upper part; 11.8 mg extract / ml methanol) and voucher specimen BW 20140717-30 (lower part) at 254 nm. Sample preparation of voucher specimen BW 20140717-30: 250 mg air dried plant powder were mixed with 250 ml methanol and sonicated for 10 min, after filtration and solvent evaporation the residue was dissolved in 2.5 ml methanol and analyzed. **LC-Method:** Agilent 1100 series HPLC system, (Agilent, Waldbronn, Germany) equipped with autosampler, column thermostat, on-line degasser, quaternary pump and DAD. Stationary phase: YMC (Kyoto, Japan) Pack Pro C18 column (150 x 4.6 mm, 3.0 µm); solvent A:water with 0.02% TFA, solvent B: acetonitrile; flow rate: 1.0 ml/min; solvent gradient: 0 min 88% A; 30.0 min to 70% A; 33 min 2% A; 45.0 min stop; post time: 10 min; column oven temperature: 45° C, injection volume: 5 µl.


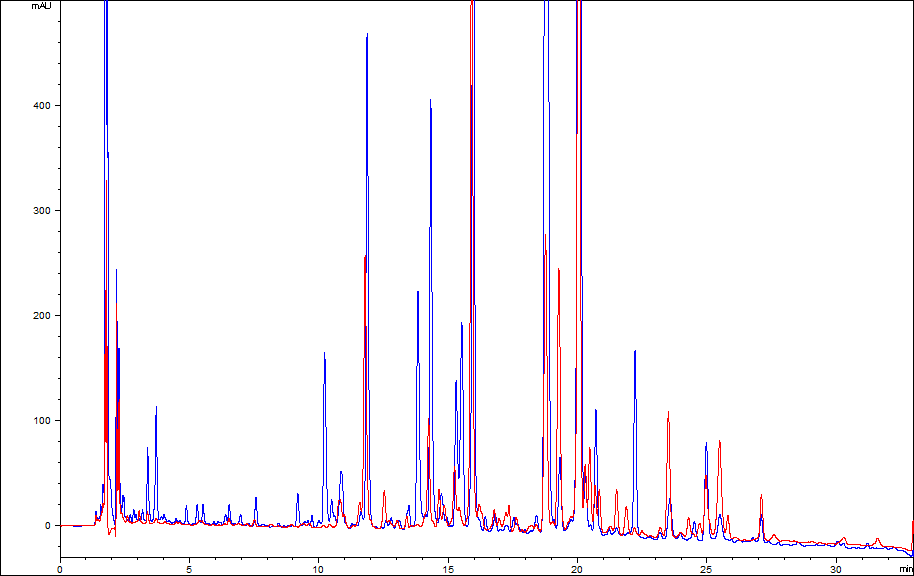


**Figure S2.** HPLC-comparison of methanolic extracts of the aerial plant parts of *W. carinthiaca* voucher specimen MD01-2319 (blue line; 11.8 mg extract / ml methanol) and voucher specimen BW 20140717-30 (red line) at 205 nm (enlarged view). LC-parameter see Figure S1.
